# Advancing Alzheimer’s disease and related dementias (ADRD) and COVID‐19 research by linking real‐world data with a standardized longitudinal ADRD data platform

**DOI:** 10.1002/alz.094785

**Published:** 2025-01-09

**Authors:** Sarah A Biber, Maria G Prado, Jessica E. Culhane, Jim Phuong, Ben Keller, Melissa Lerch, Sophia Wang, James M. Noble, Krista L. Moulder, Andrew J. Saykin, Sujuan Gao, Albert Lai, Karthik Natarajan, Walter A. Kukull, Sean Mooney, Kari Stephens

**Affiliations:** ^1^ University of Washington, Seattle, WA USA; ^2^ National Alzheimer’s Coordinating Center, University of Washington, Seattle, WA USA; ^3^ Indiana Alzheimer’s Disease Research Center, Indianapolis, IN USA; ^4^ Columbia University Irving Medical Center, New York, NY USA; ^5^ The Charles F. and Joanne Knight Alzheimer Disease Research Center, St. Louis, MO USA; ^6^ Washington University School of Medicine in St. Louis, St. Louis, MO USA; ^7^ Columbia University, New York, NY USA; ^8^ Department of Medicine, University of Washington, Seattle, WA USA

## Abstract

**Background:**

Persons with Alzheimer’s disease and related dementias (ADRD) have been disproportionally impacted by the COVID‐19 pandemic, showing a significantly increased risk of infection and severe illness, including neurocognitive consequences. The National Alzheimer’s Coordinating Center collects rich longitudinal, standardized neurocognitive data from populations at high risk of COVID‐19 complications, including older adults who were cognitively normal prior to infection and those who had pre‐existing ADRD. These data, in combination with Electronic Health Records (EHR) clinic data, will be critical for understanding the complex pathophysiology and cognitive symptoms of COVID‐19 and the development of future therapeutics. To rapidly respond to the COVID‐19 epidemic, the present study developed a novel socio‐technical solution for the Alzheimer’s Disease Research Centers (ADRC) Program to expand longitudinal COVID‐19 data collection, integrate new data streams, and open the door to impactful new COVID‐19 and ADRD discoveries.

**Method:**

We linked EHR, Medicare claims, and genetic data with rich standardized longitudinal cognitive assessment data collected from across the ADRC Program and housed at NACC. This project included the development and deployment of standardized IRB and consent language and a new data use agreement to provide access to all data steams within the LINKAGE enclave. It also leveraged an OMOP model to collect, harmonize, and integrate EHR data from participating ADRCs. The governance materials and technical pipelines developed will allow us to scale our solution across the heterogeneous consortium of 37 ADRCs.

**Result:**

The new team science model we developed facilitates collaborations between informatics leaders and ADRD leaders within and across institutions. Our approach supports development and dissemination of best practices to advance data interoperability and will facilitate researchers asking and answering new questions at the intersection of COVID and ADRD. The projected cohort size for this pilot study across three ADRCs is: cognitively normal (1,109), mild cognitive impairment (214), and dementia (224).

**Conclusion:**

Once scaled to the full ADRC Program, this platform will provide one of the largest and most comprehensive longitudinal multimodal datasets in the world for an age group at high risk for COVID‐19 infection. Additionally, the dataset includes valuable cognitive, behavioral, and genetic data collected prior to COVID‐19 infection.

